# Incidence and risk factors for post-penetrating keratoplasty glaucoma: A systematic review and meta-analysis

**DOI:** 10.1371/journal.pone.0176261

**Published:** 2017-04-21

**Authors:** Suqian Wu, Jianjiang Xu

**Affiliations:** Department of Ophthalmology and Visual Science, Eye, Ear, Nose, and Throat Hospital, Shanghai Medical College, Fudan University, Shanghai, China; University of South Alabama Mitchell Cancer Institute, UNITED STATES

## Abstract

**Objectives:**

To establish the incidence and risk factors for post penetrating keratoplasty glaucoma (PKKG).

**Methods:**

Studies published between 1947 and 2016 regarding penetrating keratoplasty (PK) were identified using an electronic search and reviewed. For search purpose, PKKG was defined as ocular hypertension (> 21mmHg) after PK. The incidence and risk factors of PKKG were extracted for all studies. Pooled incidence, odd ratios (ORs) and 95% confidence intervals (CIs) were calculated.

**Results:**

Thirty studies reporting on 27146 patients were included in the analysis of the incidence and risk factors for PKKG. Exact PKKG definitions used in the literature could be classified in to three subgroups: I, ocular hypertension (> 21mmHg) after PK; II, I plus > 4 weeks medical treatment required; III, II plus treatment escalation among patients with preexisting glaucoma. Overall (Definition I) pooled incidence in all studies was 21.5% (95% CI 17.8%, 25.7%). The incidence varied according to different definitions. The highest incidence value was found when only studies using Goldmann tonometer were included (22.5%), while the lowest incidence was found when a strict definition was used and steroid-induced PPKG was excluded (12.1%). The incidence was higher in patients with preexisting glaucoma, bullous keratopathy (BK), aphakia, pseudophakia, failed graft, and surgical indication of trauma. A triple procedure (combined PK with extra capsular cataract extraction and intraocular lens implantation) was not identified as being associated with the increased risk for PKKG.

**Conclusions:**

The overall pooled incidence of PKKG was 21.5%, but it varied according to the criteria used to define the presence of PPKG. Strong risk factors for PKKG included preexisting glaucoma and aphakia, while modest predictors included pseudophakia, regrafting, and preoperative diagnosis like BK and trauma. There may not be sufficient evidence to identify a significant association between a triple procedure and PKKG.

## Introduction

Glaucoma continues to be a frequent complication of penetrating keratoplasty (PK) and has been determined to be a primary cause of graft failure [1], with yet unexplained determinants. It has also been reported to be one of the three most common causes for graft failure along with rejection and infection [2–5]. The reported prevalence of glaucoma or ocular hypertension after PK varies remarkably [6], ranging from 5.3% [7] to 60% [8] according to the published literature, of which an important potential reason is lacking an universally recognized “gold-standard” of diagnosis. Also, the factors that contribute to clinically significant glaucoma after PK have not been fully established and are currently a matter of vital importance, which emphasizes the need for further investigation and to identify variables that can be modified to control intraocular pressure (IOP) after PK.

The objective of this meta-analysis was to identify all currently published literature to establish the incidence of post-penetrating keratoplasty glaucoma (PPKG), along with its major confounding factors, and its risk factors.

## Materials and methods

### Study selection

A systematic review of the published literature on PPKG was conducted following the Meta-Analysis of Observational Studies in Epidemiology (MOOSE)[9] and the Preferred Reporting Items for Systematic Reviews and Meta-Analyses (PRISMA) guidelines (S1 Fig) [10]. A computerized search was performed to identify all relevant studies published from January 1, 1947, to March 1, 2016, in the Medline, EMBASE and Cochrane Library database. The following search terms were used: *penetrating keratoplasty* and *corneal transplantation*. Citations were screened at the title and abstract level and retrieved as a full report if they reported on outcomes after PK. The search term “*glaucoma*” was then used in each retrieved paper to single out papers reporting data on PPKG. S2 Fig shows the search strategy used for each database. The full texts and bibliography of all potential articles were also reviewed in detail to search for additional relevant studies. In addition, a Google search was performed to locate relevant publications from national corneal transplantation registry web sites.

#### Inclusion criteria

Studies were included if the following criteria applied: 1) reported on incidence or risk factors for PPKG; 2) reported to have included consecutive patients; 3) performed a minimum of 30 PK procedures; and 4) enrollment for PK was based on existing and accepted guidelines. When two or more publications were based on the same population, the publication with a larger sample size was included in the analysis.

#### Exclusion criteria

Studies were excluded if any of the following criteria applied: 1) duplicate publication, overlap of patients, subgroup studies of a main study; 2) outcome of interests was not clearly reported or was impossible to extract or calculate from the results; and 3) a graft other than fresh donor cornea (e.g., keratoprosthesis, glycerol-preserved donor cornea) was used.

#### Definitions

For the purpose of our current search and analysis, PPKG was defined as ocular hypertension (> 21mmHg) after PK.

#### Data extraction and quality assessment

Data were independently extracted from eligible studies by two reviewers. Relevant information was collected and included but was not limited to, year and journal of publication, first author, study design, inclusion and exclusion criteria, IOP measurement methods, definition of endpoints, number of subjects included, study population demographics, follow-up period, and outcomes. The study quality was also assessed in terms of prospective study design (prospective vs. retrospective) and multivariate statistical analyses (univariate vs. multivariate logistic regression) [11].

### Statistical analysis

The proportion of individuals with PPKG in each study was logit transformed, meta-analyzed and back-transformed to obtain a pooled incidence of PPKG for all studies. The incidence of PPKG was compared according to preexisting glaucoma, bullous keratopathy (BK), aphakia, pseudophakia, triple procedure, regrafting, and surgical indications including herpes simplex keratitis (HSK) and trauma, using an odd ratio (OR) with a 95% confidence interval (CI). Data were pooled using a DerSimonian-Laird’s random-effects model to obtain a more conservative estimate of the incidence of PPKG and the odds of PPKG in these various groups [12,13]. Statistical significance was set at p < 0.05 (2-tailed). Heterogeneity was assessed by H^2^ and I^2^ test. The robustness of our findings was also analyzed by omitting one study at a time. In addition, publication bias was monitored and tested using funnel plots and fail-safe N (Rosenthal’s for incidence analysis and Orwin’s for risk factor analysis) when more than 10 studies were involved [14]. In risk factor analysis, criterion for a ‘trivial’ OR was set as 1.05, and the mean OR for missing studies was set as 1.00. If a publication bias was observed, the corresponding incidence or OR along with the CI was subsequently adjusted by Duval and Tweedie’s trim and fill [15]. Data analysis was performed using Comprehensive Meta Analysis Version 2 (Biostat Inc., Englewood, NJ), except the confidence intervals for I^2^ which were calculated manually using the formulae from Higgins *et al* [16].

## Results

Through a keywords search, 19667 reports were identified and reviewed at the title and abstract level. Further evaluation narrowed the selection to 76 potential publications. A manual search of the bibliographies identified 2 additional relevant publications. When the inclusion and exclusion criteria were applied, 31 articles remained (S1 Fig). Of the 31 publications, 30 were used for assessing the incidence of PPKG (Table 1), and 18 publications remained for assessing the risk factors of PPKG (Table 2). Two of the 31 papers came from one center; however, one was used for assessing incidence [5] and the other was used for assessing the risk factors of PPKG [6]. The studies included in the analysis were from 14 countries and were published between 1972 (when the earliest PKKG report was found) and 2015. The 30 publications included for the incidence of PPKG had 7 prospective and 23 retrospective studies, and the remaining study [6] also had a retrospective design. Seven studies used Definition III, the strictest PPKG definition, among 30 selected papers [5,17–22], and 5 articles reported an incidence of PPKG that excluded steroid-induced glaucoma [20,22–25]. Nine surveys reported early incidence of PPKG, though the definition of “early” varied from 2 days to 3 months [18,20,22–24,26–29].

**Table 1:** Selected studies on the incidence of PPKG.

First Author	Center	Year	Study design	Subjects	Mean follow-up	IOP measurement approach	PPKG definition
Sharma RA	UK	2016	Retrospective	35	24 months	Goldmann*	I
Chen	China	2015	Prospective	108	384 days	Not mentioned	II
Oruçoglu	Israel	2014	Retrospective	146	24.4 months	Goldmann	I
Yu	Germany	2014	Retrospective	377	39.3 months	Not mentioned	II
Sharma A	India	2014	Retrospective	445	32 months	Goldmann	I
Huber	Germany	2013	Retrospective	1848	24 months	Goldmann	III
Yildirim	Turkey	2011	Retrospective	122	38.9 months	Tono-Pen^†^	III
Anshu	Singapore	2011	Prospective	901	36.8 months	Not mentioned	I
Karadag	Turkey	2010	Retrospective	749	5 months	Not mentioned	III
Wagoner	Saudi	2009	Retrospective	910	Not mentioned	Not mentioned	III
Rahman	UK	2009	Retrospective	203	61 months	Not mentioned	III
Williams	Australia	2007	Retrospective	13350	> 1 year	Not mentioned	I
Patel	USA	2005	Prospective	388	Not mentioned	Not mentioned	II
Allouch	France	2003	Prospective	410	33.2 months	Goldmann	II
França	Brazil	2002	Retrospective	228	17.1 months	Not mentioned	II
Sit	Canada	2001	Retrospective	468	37.7 months	Not mentioned	I
Nguyen	Germany	2000	Retrospective	534	2.7 years	Goldmann	I
Redbrake	Germany	2000	Prospective	75	60 months	Goldmann	I
Xie	China	2000	Retrospective	1500	Not mentioned	Pneumotonometry	I
Wylegala	Poland	1999	Retrospective	537	Not mentioned	Pneumotonometry	II
Sihota	India	1998	Retrospective	747	23 months	Goldmann	III
Sekhar	India	1993	Retrospective	232	14.5 months	Goldmann	I
Chien	USA	1993	Prospective	155	1 week	Tono-Pen^†^	I
Kirkness	UK	1992	Retrospective	1122	Not mentioned	Not mentioned	III
Simmons	USA	1989	Retrospective	229	84 weeks	Goldmann	I
Foulks	USA	1987	Retrospective	502	3 years	Goldmann or Mackay-Marg^‡^	II
Karesh	USA	1983	Retrospective	80	22 months	Pneumotonometry	I
Kushwaha	India	1981	Retrospective	185	15 days	Not mentioned	I
Goldberg	USA	1981	Prospective	137	Not mentioned	Not mentioned	I
Wood	USA	1972	Retrospective	423	Not mentioned	Mackay-Marg^‡^	I

PPKG, post penetrating keratoplasty glaucoma; I, ocular hypertension (> 21mmHg) after penetrating keratoplasty; II, ocular hypertension (> 21mmHg) after penetrating keratoplasty, which needed an anti-glaucoma surgical procedure or medications to lower intraocular pressure for more than 4 weeks; III, the presence of persistent elevated intraocular pressure, above 21 mmHg or > 10 mmHg from the baseline value, that required the introduction of anti-glaucoma drops or surgical intervention at any time. In patients with pre-existing glaucoma, PPKG was defined as uncontrolled intraocular pressure with the original treatment regime that required escalation of treatment after penetrating keratoplasty.

* Goldmann applanation tonometry.

^†^ Tono-Pen tonometry.

^‡^ Mackay-Marg electronic applanation tonometer.

**Table 2:** Included studies on the risk factors for PPKG.

First Author	Subjects	Regression Model	Included on the risk factors for PPKG							
			Preexisting glaucoma	BK	Aphakic*	Pseudophakic*	HSK	Trauma	Regraft	Triple procedure^†^
Oruçoglu	146	M	+							
Sharma A	445	M	+	+						+
Yildirim	122	M	+	+						
Karadag	749	U	+	+	+	+	+	+	+	
Rahman	203	U		+					+	
Al-Mohaimeed	715	M	+	+	+	+			+	
Allouch	410	U		+					+	
França	228	U		+			+	+	+	
Nguyen	534	U								+
Redbrake	75	U	+							
Sekhar	232	U		+	+	+			+	+
Sihota	747	U		+	+	+			+	
Chien	155	M	+							+
Kirkness	1122	U		+			+	+		
Simmons	229	U	+		+	+				
Foulks	502	U	+		+	+				
Karesh	80	U	+		+					
Goldberg	137	U		+			+	+	+	

PPKG, post penetrating keratoplasty glaucoma; BK, bullous keratopathy (either aphakic or pseudophakic); HSK, herpes simplex keratitis; U, univariate regression medel; M, multivariate regression model.

* Either preoperative or at-graft.

^†^ Penetrating keratoplasty + extra capsular cataract extraction (ECCE) + intraocular lens (IOL) implantation.

An analysis of the incidence was performed on 27146 patients. The sample size varied from 35 [8] to 13350 [30] (Table 1). Indications for PK were collected (see Appendix 1). The IOP was measured by Goldmann applanation tonometry in 5203 patients (19.2%) of 11 publications and was measured by the Tono-Pen tonometry in 277 patients (1.0%) in 2 studies. Two studies used Mackay-Marg electronic applanation tonometer in 925 subjects (3.4%). In addition, another three studies used pneumotonometry to measure IOP in 2153 subjects (7.9%). The IOP measurement approach was not mentioned in the remaining 19126 patients (70.5%).

The exact definition of PPKG varied among the selected studies. Basically, they could be classified into three subtypes:

1. Ocular hypertension (> 21mmHg) after PK.
2. Ocular hypertension (> 21mmHg) after PK (I), which needed an anti-glaucoma surgical procedure or medications to lower IOP for more than 4 weeks.
3. The presence of persistent elevated IOP above 21 mmHg or > 10 mmHg from the baseline value that required the introduction of anti-glaucoma drops or surgical intervention at any time, with or without visual field loss and/or optic nerve changes. In patients with pre-existing glaucoma, PPKG was defined as uncontrolled IOP with the original treatment regime that required an “escalation of treatment” after PK.

**Fig 1:**
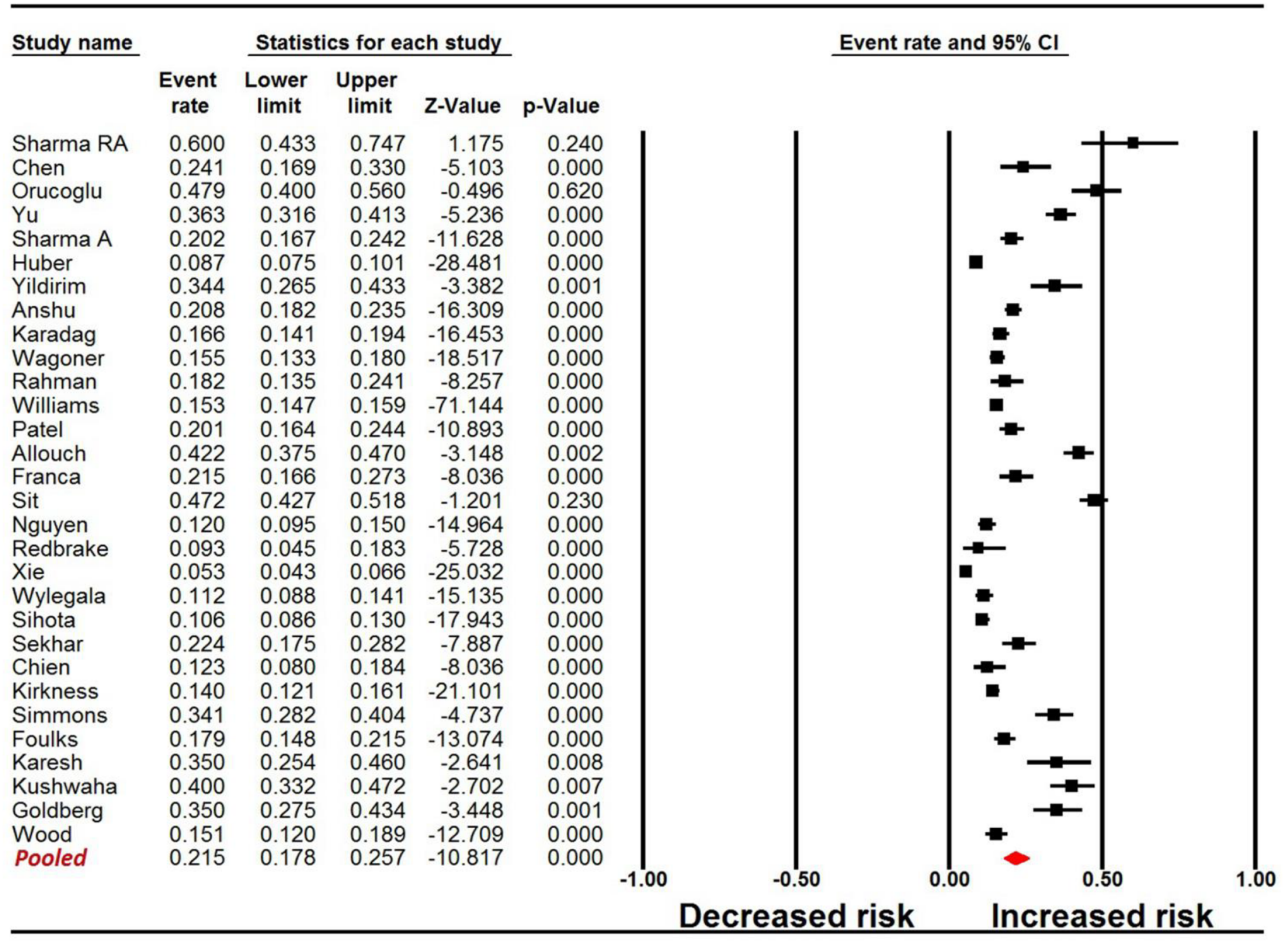
Forest plot for the pooled incidence of PPKG Definition I. The pooled estimate for the overall incidence (Definition I) of PPKG was 21.5% (95% CI: 17.8 to 25.7). PPKG, post-penetrating keratoplasty glaucoma.

The pooled estimate for the overall incidence of PPKG was 21.5% (95% CI: 17.8 to 25.7, I^2^ = 97.4%; Table 3, Fig 1). The value varied according to different filtering criteria (Table 3), including the three definitions that were presented previously. The lowest incidence reported was 5.3% in a study from China using the Definition I criteria, while the highest incidence was 60%, which was reported in a United Kingdom survey that used the Definition I criteria. In addition, the incidence was similar between prospective and retrospective study groups.

**Table 3:** Incidence of PPKG according to various filters.

Filtering criteria	Number of studies	Number of subjects	Pooled incidence (%)	95% CI (%)	H^2^	I^2^ (%)	95% CI of I^2^ (%)
All (Definition I, II & III)	30	27146	21.5	17.8, 25.7	38.5	97.4	95.6, 98.4
Definition II & III	14	8251	19.2	14.4, 25.1	31.7	96.9	93.1, 98.5
Definition III	7	5701	15.4	11.7, 20.0	13.7	93.7	75.4, 97.9
Early incidence	9	5149	11.9	7.6, 18.4	23.3	95.7	87.5, 98.5
Steroid-induced PPKG excluded	5	3464	17.4	11.5, 25.6	22.2	95.5	78.5, 97.9
Definition III & Steroid-induced PPKG excluded	2	2597	12.1	6.3, 22.0	29.4	96.6	-
Prospective	7	2174	22.4	15.5, 31.3	17.2	94.2	80.2, 98.2
Retrospective	23	24972	21.3	17.1, 26.1	41.7	97.6	95.7, 98.7
**Using Goldmann Tonometer**	11	5203	22.5	14.7, 32.8	43.5	97.7	94.2, 99.1

PPKG, post penetrating keratoplasty glaucoma; CI, confident interval; Definition I, ocular hypertension (> 21mmHg) after penetrating keratoplasty; Definition II, ocular hypertension (> 21mmHg) after penetrating keratoplasty, which needed an anti-glaucoma surgical procedure or medications to lower intraocular pressure for more than 4 weeks; Definition III, the presence of persistent elevated intraocular pressure, above 21 mmHg or > 10 mmHg from the baseline value, that required the introduction of anti-glaucoma drops or surgical intervention at any time. In patients with pre-existing glaucoma, PPKG was defined as uncontrolled intraocular pressure with the original treatment regime that required escalation of treatment after penetrating keratoplasty.

Our search for predictors of PPKG identified 18 publications on 8 major **factors** (Table 4). The pooled incidence was much higher in those with preexisting glaucoma and in aphakic individuals, and was slightly higher in those with BK, pseudophakia, regrafting and preoperative diagnosis as trauma. The combination procedure of PK with extra capsular cataract extraction (ECCE) + intraocular lens (IOL) implantation seemed to be insignificantly associated with the increasing incidence of PKKG. Notably, the results remain similar when only high quality (using multivariate regression) studies were included. The pooled OR for trauma with PPKG became insignificant on removal of the studies by Kirkness et al [17]. Nevertheless, there was a trend toward an increased incidence.

**Table 4:** Association of various factors with PPKG according to pooled OR.

Risk factor	Number of studies	Number of subjects	Pooled OR (95% CI)	H^2^	I^2^ (%)	95% CI of I^2^ (%)	Removed study that made pooled OR insignificant
Preexisting glaucoma	10	3214	5.50 (2.86, 10.58)	6.17	83.8	56.9, 93.9	None
-Studies using multivariate model	5	1583	3.50 (1.78, 6.74)	2.85	64.9	-	None
BK*	11	5195	2.00 (1.46, 2.74)	3.38	70.4	25.3, 88.2	None
-Studies using multivariate model	3	1282	1.90 (1.19, 3.02)	1.82	45.0	-	None
Aphakia*	8	3254	4.23 (2.78, 6.43)	3.00	66.7	-	None
Pseudophakia*	6	3164	1.56 (0.93, 2.61)	4.41	77.3	10.2, 94.2	None
HSK	4	2388	1.08 (0.49, 2.35)	1.45	30.9	-	-
Trauma	4	2234	2.62 (1.21, 5.68)	2.39	58.2	-	Kirkness et al.
Regraft	8	3487	1.83 (1.05, 3.19)	4.27	76.6	27.0, 92.5	None
Triple procedure^†^	4	1366	0.92 (0.37, 2.28)	5.15	80.6	-	-
-Studies using multivariate model	2	600	0.99 (0.20, 4.84)	5.78	82.6	-	-

PPKG, post penetrating keratoplasty glaucoma; OR, odd ratio; CI, confidence interval; BK, bullous keratopathy (either aphakic or pseudophakic); HSK, herpes simplex keratitis.

* Either preoperative or at-graft.

^†^ Penetrating keratoplasty + extra capsular cataract extraction (ECCE) + intraocular lens (IOL) implantation.

**Fig 2:**
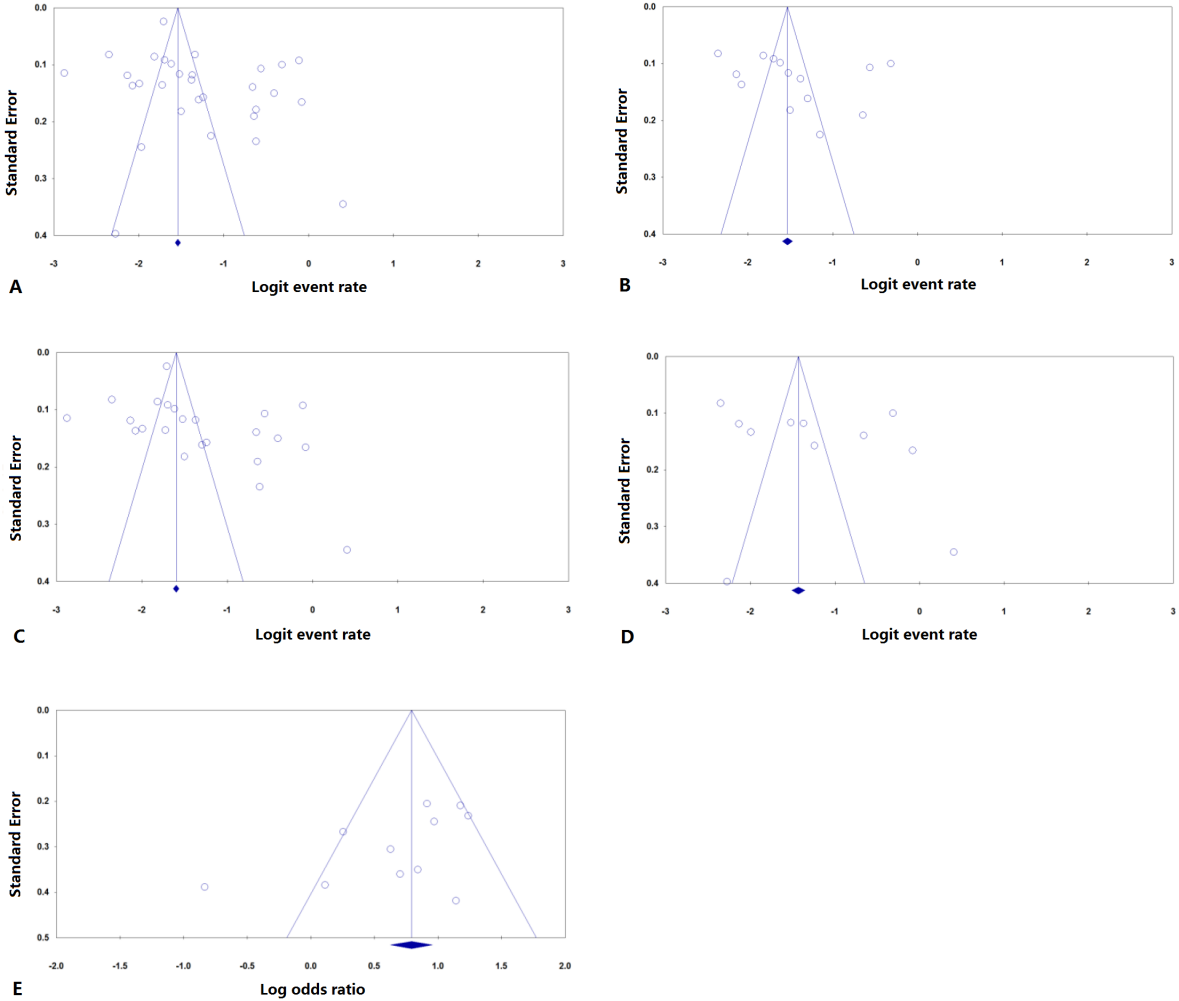
Funnel plots of publication bias analyses for PPKG incidence (A-D) and a risk factor (E, BK). Incidences filtered by Definition I (A), Definition II (B), retrospective studies only (C) and studies using Goldmann tonometer (D) were tested for potential publication bias. PPKG, post penetrating keratoplasty glaucoma; BK, Bullous keratopathy.

Table 5 and Fig 2 showed the publication bias analyses for all that included more than 10 studies. All the fail-safe N values remained considerably large except that for BK which was a little smaller but still large. In addition, analysis for BK was the only one that had a pooled value change under Duval and Tweedie’s trim and fill, suggesting that a publication bias might exist and a more “real” OR might be close to 1.87. Nevertheless, this “real” value was very close to what was calculated.

**Table 5:** Publication bias analysis.

Study Criteria		Number of Studies	Fail-safe N	Pooled value (95% CI)	Pooled value (95% CI) adjusted with Duval and Tweedie’s trim and fill
Incidence	All (Definition I)	30	84130*	21.5 (17.8, 25.7)^‡^	21.5 (17.8, 25.7) ^‡^
	Definition II	14	7937*	19.2 (14.4, 25.1)^‡^	19.2 (14.4, 25.1) ^‡^
	Retrospective	23	24927*	21.3 (17.1, 26.1)^‡^	21.3 (17.1, 26.1) ^‡^
	Using Goldmann Tonometer	11	2965*	22.5 (14.7, 32.8)^‡^	22.5 (14.7, 32.8) ^‡^
Risk Factor	BK	11	168^†^	2.00 (1.46, 2.74)^§^	1.87 (1.36, 2.57)^§^

BK, bullous keratopathy (either aphakic or pseudophakic)

* Rosenthal’s fail-safe N

^†^ Orwin’s fail-safe N

^‡^ Pooled incidence (%)

^§^Pooled Odd Ratio

## Discussion

This systematic review and meta-analysis included data from all available and identified studies that reported the incidence of PPKG. The reported incidence varied strikingly, and, as was mentioned, there is no “gold standard” of PPKG diagnosis. As was found in our results, the PPKG definition, no matter I, II or III, was significantly distinguished from the classic definition of glaucoma. A major potential reason may be the widely-acknowledged difficulty to perform visual field assessment and fundus imaging for post-PK patients. In our study, the definition criteria used were found to lead to the different incidences, which was lowest when Definition III criteria were used and highest with a broad definition of PPKG (Definition I). Because Definition III is the strict and universally acknowledged PPKG definition, it could be inferred that the real incidence of PK-induced glaucoma might be close to 15.4%. In terms of the exclusion of steroid-induced PPKG, the incidences were 17.4% and 12.1% in all patients and in those that fit the Definition III criteria; however, among the PPKG patients of the two groups, the proportions of those without steroid-induced PPKG remained similar (77.7% and 78.6%), which suggested that the use of steroids after PK might contribute to a 21–22% proportion of intraocular hypertension or PPKG. In addition, early incidence of PPKG was approximately half of the overall incidence. Although the definition of “early” varied in the studies, it can still be inferred that PPKG occurs in different time periods according to diverse pathogenic factors and that almost half of PPKG cases may arise in the early period after PK.

According to the definitions used in this study, an IOP of 21mmHg was set as a critical value; however, regarding the extracted data, IOP was measured using different methods with diverse sensitivity and reliability. Moreover, Goldmann applanation tonometry has been considered the ‘‘gold standard” in IOP measurement, which may explain the reason that this device was most frequently used among the reported IOP measurement approaches, but it may provide inaccurate results in an edematous cornea or in a corneal transplant [31]. In fact, animal studies and ex vivo systems have suggested less accuracy with Goldmann applanation tonometry, whereas MacKay-Marg and Tono-Pen tonometers in corneas with edema and corneal transplants have been more reliable [31,32]. Therefore, the IOP could be measured with biases after PK, due to the diverse accuracy and reliability of the methods adapted, thus affecting PPKG identification.

The results of our study showed that preexisting glaucoma seemed to be a leading cause of PKKG. Previous studies indicated that preexisting glaucoma was also a major risk factor for graft failure [33,34]. Hence, IOP control is important before PK in this patient population. However, PPKG may still occur even when glaucoma was medically controlled preoperatively [29]. A particularly significant association of BK with PKKG was also found. As BK was the leading indication for keratoplasty in many countries and the majority of the pseudophakic and aphakic cases were associated with BK, it is not suitable to consider pseudophakia and aphakia as independent risk factors [21]. Probable explanations for the increased PKKG incidence in these cases include IOL material, inflammatory response after PK, the effects of aphakia and pseudophakia on the peripheral anterior angle structures, and the synechia formation [21,35].

A moderate association between regrafting and PPKG was indicated, which may attribute to a strong inflammatory response and poor peripheral anterior angle structures after PK. Surgical indication of trauma was also found to be related to more prevalent incidence of PKKG. However, as the robustness of the analysis was suggested, this result should be conservatively interpreted.

Previous study results regarding the association of a triple procedure (combined PK with ECCE and IOL implantation) with PKKG have aroused controversy [1]. Our results suggested that there was no strong evidence to prove this relevance. As phacoemulsification has predominated in industrialized countries [36], the combination of PK with phacoemulsification and IOL implantation has, therefore, been widely used in these areas [37]. Thus, the association of this combined procedure with PKKG should also be studied in the future.

The aetiology and pathogenesis of PKKG are multifactorial and complicated, causing some potential risk factors to remain undiscovered. Olson and Kaufman identified several other variables that could possibly alter the anterior chamber angle and thus increase the IOP using a mathematical model, including tight suturing, long suture bites, and same-sized donor-recipient trephination [38]. Due to our inclusion and exclusion criteria and limited article selection, these factors were unable to be meta-analyzed in this study. However, they are potential predictors of PKKG that should also be considered pre- and postoperatively.

High heterogeneity between studies was observed in our results. The reasons for the heterogeneity are speculative but may include subtle differences in the way diagnostic criteria for PPKG were defined, or in other cultural differences, including ethnicity, which were not possible to examine using the available data. Also, the large objective number is also directly linked with high heterogeneity [39]. We performed several sensitivity analyses based on various criteria (Tables 3 & 4), and found that the results remain similar, which suggests the reliability of our analyses. Although heterogeneity may be seen as precluding the pooling of data from these studies in a meta-analysis, we feel that the summary data obtained using our approaches are useful in viewing the incidence and predictors of PPKG from an epidemiological and global perspective.

This study has several strengths. An exhaustive search strategy was used to maximize the likelihood of identifying all pertinent literature. Eligibility and data extraction was judged independently by two investigators, and discrepancies were resolved by consensus. We included data from eligible foreign language papers to be as inclusive as possible. A random effects model was used to pool data in order to provide a more conservative estimate of the incidence of PPKG and was assessed for publication bias. Finally, we limited studies to those with consecutive patients and excluded those conducted with < 30 sample sizes, meaning that the likelihood that the incidence of PPKG was inflated has been minimized.

This study also has some limitations. All studies included were single-centered, except the Australian Corneal Graft Registry Report [30], decreasing the generalizability and increasing the likelihood of environmental and socio-economic bias. From the global perspective, the absence of studies that have reported on the incidence and risk factors for PKKG for some geographical regions, such as Africa and Central America, is another limitation for the generalizability of this meta-analysis. In addition, as mentioned, diverse IOP-measuring methods contributed to PPKG identification bias.

The findings of this study have implications for both clinical practice and future research. Studies using the Definition III criteria to define PKKG remain scarce, although these criteria have been used and published since 1992 [17]. Extracting and analyzing study data on the incidence of PPKG has emphasized the magnitude of this complication after PK and thus the implications for health services worldwide. Our findings also revealed the importance of determination of corticosteroid dosing in addition to anti-glaucoma management for treating PKKG and strengthened the consensus that tapering off corticosteroid and using “substitutes”, e,g., cyclosporine A, after PK seemed to be a recommendable choice [40]. Moreover, as the third most common cause for graft failure after PK, the incidence and predictors of PPKG in some geographical regions, such as Africa and Central America, need further study.

### Conclusions

This systematic review and meta-analysis has demonstrated an incidence of PPKG of almost 21.5%, but this varied, considerably in some instances, according to the diagnostic criteria and other factors such as IOP measurement methods, corticosteroid use, and follow-up time after PK. Strong risk factors for PKKG included preexisting glaucoma and aphakia, and minor risk factors included pseudophakia, regrafting, and preoperative diagnosis as BK and trauma. In addition, there may not be sufficient evidence to identify a significant association between a triple procedure and the increased incidence of PKKG.

## Supporting information

S1 Fig Preferred reporting items for systematic reviews and meta-Analyses (PRISMA) checklist. (TIFF)

S2 Fig Flow chart showing search strategy used for each database. (TIF)

S1 File Indications for PK from selected studies. (DOC)
