# Supplementary figures and images for: Incidence and risk factors for post-penetrating keratoplasty glaucoma: A systematic review and meta-analysis
Source: PLoS One. 2017 Apr 21;12(4):e0176261. doi: 10.1371/journal.pone.0176261 (PMC5400257; doi:10.1371/journal.pone.0176261)

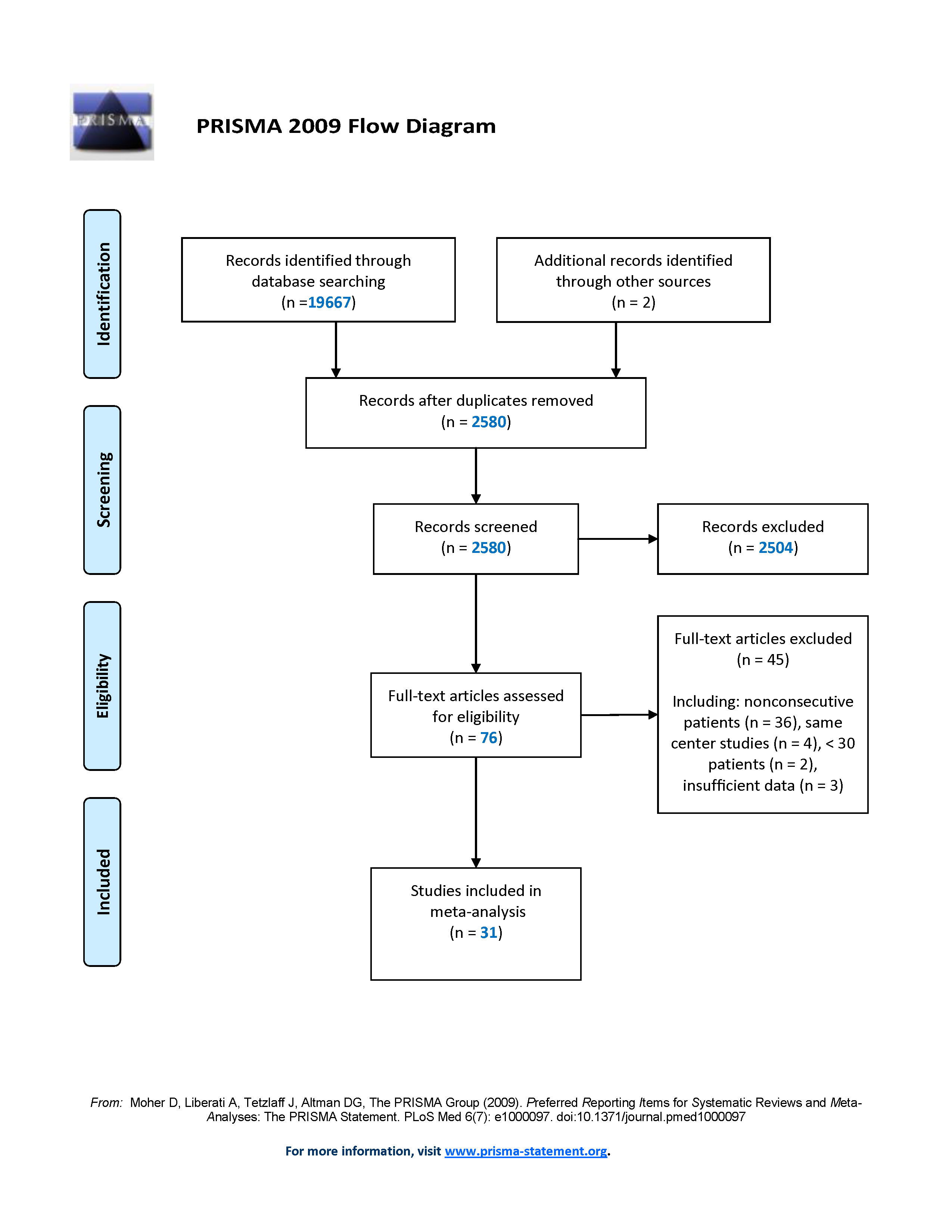

Supplement: S1 Fig — (TIFF) [file pone.0176261.s001.tiff]
