# Supplementary material for: Incidence and risk factors for post-penetrating keratoplasty glaucoma: A systematic review and meta-analysis
Source: PLoS One. 2017 Apr 21;12(4):e0176261. doi: 10.1371/journal.pone.0176261 (PMC5400257; doi:10.1371/journal.pone.0176261)
Supplement: S1 File — (DOC) [file pone.0176261.s003.doc]

S1 File. Indications for PK from selected studies

Adherent leucoma

Corneal destrophy

Bullous Keratopathy

Opaque graft

Corneal opacity

Bacteria, fungal, and viral keratitis

Corneal degeneration

Keratoconus

Iridocorneal endothelial (ICE) syndrome

Trauma

Corneal ulcers

Congenital aniridia

Limbal stem cell insufficiency

Ocular pemphigoid

Atopic keratokonjunctivitis

Silicon keratopathy

Vascularized corneal scar

Descemetocele

Chemical burn

Radiation keratitis

Corneal perforation

Mucopolysaccharidosis

High myopia in a graft
